# Virtual reality-assisted psychological treatment of aggression for violent offenders: a pilot descriptive case series in maximum-security forensic psychiatry

**DOI:** 10.3389/frhs.2026.1920018

**Published:** 2026-09-29

**Authors:** Fernando Renee González Moraga, Sean Perrin, Pia Enebrink, Kristina Sygel, Wim Veling, Märta Wallinius

**Affiliations:** 1 Evidence-based Forensic psychiatry, Department of Clinical Sciences Lund, Psychiatry, Lund University, Lund, Sweden; 2 Centre for Ethics, Law and Mental Health, Institute of Neuroscience and Physiology, The Sahlgrenska Academy at the University of Gothenburg, Gothenburg, Sweden; 3 Research Department, Regional Forensic Psychiatric Clinic Växjö, Växjö, Sweden; 4 Department of Psychology, Lund University, Lund, Sweden; 5 Division of Psychology, Department of Clinical Neuroscience, Karolinska Institutet, Stockholm, Sweden; 6 Department of Forensic Psychiatry, National Board of Forensic Medicine, Stockholm, Sweden; 7 Department of Psychiatry, University Medical Center Groningen, University of Groningen, Groningen, Netherlands

**Keywords:** aggression, forensic psychiatry, virtual reality, VRAPT, VR-assisted interventions

## Abstract

**Background:**

Aggressive behaviour remains a major clinical challenge in forensic psychiatric care, and effective interventions targeting aggression are limited. Virtual Reality Aggression Prevention Treatment (VRAPT) is a manualized cognitive behavioural intervention that uses immersive virtual reality to provide individualized exposure to emotionally provocative interpersonal situations. Following a previous randomized controlled trial in Dutch forensic psychiatric care, the VRAPT protocol was revised to strengthen its theoretical foundation and therapeutic content. This pilot descriptive case series provides the first clinical description of the revised VRAPT protocol in patients receiving treatment in a Swedish maximum-security forensic psychiatric setting.

**Methods:**

A pilot descriptive case series was conducted in a maximum-security forensic psychiatric clinic. Fourteen patients initially consented to participate, of whom seven completed the intervention and follow-up assessments. The revised VRAPT protocol emphasized therapist-guided, individualized virtual reality scenarios targeting emotion regulation during emotionally provocative interpersonal situations. Clinical, psychological, behavioural, and process measures were collected to characterize the study population and describe individual trajectories during treatment and follow-up.

**Results:**

Participants who completed treatment generally reported moderate to high levels of perceived presence, suggesting that the virtual environments were experienced as immersive and sufficiently realistic. Individual trajectories varied across participants. Several participants showed favourable changes in measures of emotion regulation, self-reported anger, and aggression, whereas others showed stable or fluctuating trajectories over time. Staff-rated aggressive behaviour also varied considerably across participants. Exploratory group-level analyses did not indicate statistically significant changes. A 50% dropout rate highlighted the challenges associated with treatment engagement and retention in a maximum-security forensic psychiatric setting. The observations were consistent with the theoretical rationale underlying VRAPT, although the uncontrolled design precludes conclusions regarding treatment effectiveness.

**Conclusions:**

This pilot descriptive case series provides the first detailed clinical description of the revised VRAPT protocol in Swedish maximum-security forensic psychiatry. The findings provide descriptive clinical observations that may inform the design of future controlled studies, including randomized controlled trials and high-quality single-case experimental designs.

## Introduction

Aggressive and violent behavior represents a persistent clinical challenge within forensic psychiatric services. In Sweden, approximately 300 individuals are sentenced each year to forensic psychiatric care for committing serious offences, most commonly involving aggressive acts such as assault, homicide or manslaughter, unlawful threats, or arson, while under the influence of a severe mental disorder (SMD) (1, 2). As in many other jurisdictions, Swedish legislation recognizes that individuals who commit crimes in the context of an SMD require specialized psychiatric care rather than traditional incarceration (3, 4). Within this legal framework, mechanisms such as the Special Discharge Review (SDR) apply to individuals assessed as posing a continued risk of serious reoffending, whereby decisions regarding leave or discharge cannot be made solely by the treating psychiatrist but must instead be determined by a court of law. This structure underscores the central role of risk assessment and violence prevention within forensic psychiatric care.

Empirical research consistently demonstrates that individuals with SMD are at significantly increased risk of engaging in aggressive behaviour compared to the general population (5–7). At the same time, this population is disproportionately vulnerable to victimisation, highlighting a dual burden of perpetration risk and exposure to violence (8, 9). Together, these clinical, legal, and epidemiological factors contribute to a complex treatment context in which the management of aggression is critical for patient rehabilitation, staff safety, and the stability of the therapeutic milieu.

The forensic populations, predominantly male, often present with long-standing histories of violence, severe mental illness, reduced empathy, impaired emotion regulation, and difficulties with impulse control (10–13). Among female forensic patients, aggression is often associated with borderline personality disorder, impulsivity, self-harm, and extended hospital stays (14). Increasing evidence suggests that emotion dysregulation constitutes a transdiagnostic mechanism underlying aggressive behaviour in forensic psychiatric populations (15, 16). Difficulties in identifying, understanding, and regulating emotional responses have been associated with institutional aggression, violent offending, psychopathic traits, impulsivity, and elevated violence risk among forensic patients. Consequently, interventions targeting emotion regulation may address an important modifiable risk factor for aggression and violence within forensic psychiatric care.

Managing aggression in these settings is crucial to ensuring the safety of both staff and residents, and to reducing the risk of violent recidivism following discharge. However, current evidence-based interventions targeting aggression in forensic psychiatric care remain limited. Existing treatments frequently demonstrate modest effect sizes, and their effectiveness is constrained by ethical and practical limitations, particularly the inability to safely expose patients to real-life provocative situations that are essential for learning regulation strategies without compromising the safety of patients, staff, and others (17, 18). As a result, there is a well-documented need for novel, effective, and ethically sound interventions designed specifically to address aggression in this population (19, 20).

In modern psychological theory, aggression is defined as a deliberate and observable behavior, whether verbal or physical, intended to cause harm to another individual or object, or to express anger (21). For a behavior to be classified as aggressive, the aggressor must believe that the act will cause harm, and the target must be motivated to avoid that harm (22, 23). Aggressive behaviors occur along a continuum ranging from relatively minor acts, such as pushing or verbal insults, to more extreme forms of violence, including serious physical harm and even lethal outcomes such as manslaughter (7). Gender differences in aggression are well-documented across developmental and cultural contexts. Men are more likely than women to engage in physical and varied forms of aggression and are also more likely to be victims of it (24–27). Young men, particularly during adolescence and early adulthood, are disproportionately involved in high-risk aggressive behaviors, a pattern often referred to as the “young male syndrome” (28–30). In contrast, aggression among women has been described as a mechanism of intrasexual competition, defense, or control within intimate relationships, with common targets including both male partners and female rivals (31). Given the multifactorial origins and serious consequences of aggression in forensic psychiatric populations, the search for effective, scalable, and context-sensitive treatment approaches remains urgent.

Recent research has highlighted the potential of immersive Virtual Reality (VR) as a valuable tool in clinical practice, particularly within forensic psychiatry (32, 33). VR has been proposed as a complementary method for diagnostic assessment and psychological treatment, offering new possibilities for engaging hard-to-reach populations and enhancing treatment efficacy (34–36). Its immersive nature allows individuals to experience controlled, yet realistic, scenarios that simulate everyday social interactions. VR thereby bridges the gap between traditional therapy, laboratory research, and real-life behavior, increasing ecological validity and patient motivation (37–41).

A promising application of VR in forensic psychiatry is Virtual Reality Aggression Prevention Training (VRAPT), a manualized intervention specifically developed to help individuals with severe aggression problems. During VRAPT, patients are immersed in individualized virtual interpersonal scenarios tailored to their own aggression triggers and treatment goals. The therapist controls the scenarios in real time, gradually adjusting the intensity and social interactions to match the patient's emotional responses and therapeutic progress. Patients are encouraged to identify early emotional, cognitive, physiological, and behavioural signs of escalating aggression while practicing alternative coping strategies, perspective-taking, communication skills, and behavioural responses. Sessions include preparation before entering the virtual environment, guided therapeutic work during the VR exposure, and structured reflection afterwards to consolidate learning and relate the experiences to real-life situations (19, 42, 43). The intervention draws on principles from cognitive behavioral therapy (CBT) and is theoretically grounded in the General Aggression Model [GAM; (23, 44)], evolutionary psychology (25, 45), and evidence-based practices from forensic and clinical settings (21, 36, 46–48). In this regard, immersive technologies such as VRAPT have emerged as promising tools. Through personalized exposure to provocative scenarios and real-time physiological feedback, VRAPT allows individuals to practice emotion regulation, perspective-taking, and social problem-solving in ways that are difficult or impossible to simulate in real life (19, 42, 43).

A multicenter randomized controlled trial of VRAPT involving 128 forensic psychiatric patients was carried out in the Netherlands (43). Although the intervention did not produce significant reductions in self-reported or staff-observed aggressive behaviours, participants demonstrated improvements in hostility, anger control, and non-planning impulsiveness. However, these improvements were not maintained at three-month follow-up. Building on these findings, the VRAPT protocol was subsequently revised to strengthen its theoretical framework and therapeutic rationale (19). Rather than fundamentally changing the intervention, the revised protocol reconceptualized VRAPT as a manualized cognitive behavioural treatment grounded in the General Aggression Model (GAM), thereby providing a broader framework for understanding how individual vulnerabilities, situational factors, emotional processes, and cognitive appraisals interact to influence aggressive behaviour. The revised protocol also placed greater emphasis on emotion regulation and incorporated corresponding modifications throughout the treatment modules while retaining individualized virtual reality role-play as the core therapeutic component. The present pilot descriptive case series study provides the first detailed clinical description of the revised VRAPT protocol in a maximum-security forensic psychiatric setting, with the aim of informing future controlled evaluations. Importantly, VRAPT addresses a major limitation of traditional treatments by enabling safe, repeated exposure to emotionally charged interpersonal situations, tailored to the individual's personal triggers and learning needs (19, 36).

VRAPT has not yet been described within the Swedish forensic psychiatric system. Given the high prevalence of aggression among patients sentenced to care under the Swedish Forensic Mental Care Act, and the legal constraints placed on discharge and leave decisions (1, 2), there is a pressing need to explore interventions targeting aggression and emotion regulation in this population. Individuals in this system often suffer from severe mental disorders and demonstrate higher rates of aggressive behavior and violent victimization compared to the general population (5, 6, 9), making targeted treatment both clinically and ethically imperative. As the revised VRAPT protocol has not previously been described in routine forensic psychiatric practice, an initial clinical description may provide valuable insights into treatment delivery, individual clinical trajectories, and practical considerations that can inform future controlled evaluations.

## Aim

The purpose of the present study was to conduct a pilot descriptive case series study of the revised Virtual Reality Aggression Prevention Training (VRAPT) protocol in a Swedish forensic psychiatric setting, as developed and described in Tuente, Wallinius, González Moraga, and Veling (49) (see section on fidelity in the VRAPT intervention). The broader goal was to gather clinical observations and generate hypotheses necessary to inform the design of future randomized controlled trials and high-quality single-case experimental studies. The aim of the present study was to describe the clinical application of the revised VRAPT protocol in a maximum-security forensic psychiatric setting. This included describing patient engagement, adherence to the intervention schedule, and the overall tolerability of the treatment process. In addition, the study descriptively examined patterns in the occurrence and severity of aggressive behaviors, based on weekly staff observations over the course of the 12 weeks before, during, and 12 weeks after the intervention. A range of baseline clinical, psychological, and process measures was also collected to characterize the study population, describe intervention-related processes (e.g., presence in the virtual environment), and generate hypotheses regarding individual differences and potential mechanisms that may inform the design of future randomized controlled trials.

## Methods

A total of 14 inpatients initially agreed to participate, of whom seven (50%) withdrew from the study. Three withdrew before initiating treatment, and four withdrew after treatment had commenced. Reasons for drop-out cannot be specified due to ethical regulations stating that participants do not need to provide this information when dropping out, but it is noted that personal reasons (*n* = 4) and treatment-related/logistical reasons (*n* = 3) were rather similarly distributed. The final, seven participants included one woman and six men, with a mean age of 36 years (SD = 10.5, range = 22–46), recruited from a maximum-security forensic psychiatric clinic in Sweden. Inclusion criteria were: 1) ongoing treatment under the law of forensic psychiatric care, and 2) history of aggression and current problems with reactive aggression. Results will be reported only for the participants who completed their participation. This study was conducted as part of a larger mixed-methods research program, with qualitative findings reported elsewhere [see (50–52)]. For organization of data and generation of descriptive visual representations, IBM SPSS Statistics 29 and R Project version 4.4.2 were employed. The results are presented in individual case graphs. Given the descriptive nature of this pilot case series and the small sample size, inferential analyses were considered exploratory and were included only to complement the descriptive presentation of individual clinical trajectories. Accordingly, an exploratory Wilcoxon signed-rank test was conducted to examine changes in Social Dysfunction and Aggression Scale–9 (SDAS-9) scores from pre-treatment to follow-up.

## Procedure

### Ethical considerations

The present study, as part of a VRAPT pilot study, was approved by the Swedish Ethical Review Authority (Dnr: 2019-02337; 2020-06317) and all participants provided their written consent to participate in the study. Ethical sensitivity was of particular importance given the involvement of forensic psychiatric patients, a population considered especially vulnerable due to the combination of mental illness and legal coercion. Participation in research within such settings entails heightened ethical demands regarding autonomy, voluntariness, and protection from undue influence. The coercive nature of forensic care environments may create a perceived pressure to comply with research procedures, underscoring the importance of clear communication and genuine informed consent. All participants received both written and verbal information about the study's aims, procedures, and their rights, including the assurance that participation was entirely voluntary and that withdrawal could occur at any stage without affecting their ongoing treatment or legal status. Written informed consent was obtained prior to participation. To safeguard confidentiality and minimize risks, all collected data were pseudonymized before analysis, and identifying information was removed. Data were stored securely on password-protected computers accessible only to the research team. No information that could directly or indirectly identify individual participants was reported. Given the participants’ restricted autonomy and dependence on institutional structures, the study was designed to ensure that no additional burden, risk, or interference with clinical routines occurred as a result of participation. The study adhered to the ethical principles of respect for persons, beneficence, and justice.

### Participants and recruitment

Patients were eligible for participation if they (a) were sentenced to forensic psychiatric care and receiving treatment at a ward within the forensic psychiatric clinic where the study was conducted, and (b) had an identified need for treatment targeting aggression-related problems, such as a history of aggression and current problems with reactive aggression. Exclusion criteria were (a) inability to understand the study information and provide informed consent, as assessed by the responsible psychiatrist; (b) insufficient proficiency in spoken or written Swedish to allow active participation; (c) epilepsy; (d) intellectual disability (IQ < 70); (e) autism spectrum disorder associated with severe functional impairment; (f) an acute psychotic state; or (g) being considered by the treating psychiatrist or the clinic's security manager to pose a serious security risk that would prevent active participation, for example, severe violent behaviour that could not be safely managed during treatment.

Health professionals working at the current clinic were informed about the study by research staff, including the study objectives, eligibility criteria, and referral procedures. Eight treating psychiatrists, responsible for the clinical care of patients on the participating wards, were involved in identifying and referring potentially eligible patients under their care. Following referral, the research staff informed (verbally and in writing) the eligible participants about the study and their opportunity to participate. After informed consent was obtained, participants were enrolled in the study. Data were collected at five points (see Table 1 and Measures for details):

- T0 (Baseline): 12 consecutive weeks before the intervention. Data consisted of staff observations using SDAS-9 and clinical/sociodemographic data from medical records.
- T1 (Pre-intervention): Participant self-reports using CTQ-SF, DERS, STAXI-2, AQ-RSV, and ESI-BF.
- T2 (Intervention): Staff observations (SDAS-9) were collected continuously during the VRAPT sessions.
- T3 (Post-intervention): Participant self-reports (DERS, STAXI-2, AQ-RSV, IPQ) and qualitative interviews with participants and VRAPT therapists [see (50, 52)].
- T4 (Follow-up): 12 consecutive weeks after the intervention, including staff observations (SDAS-9) and participant self-reports (DERS).

**Table 1:** Overview of data collection timeline. The table illustrates the five time points of data collection. T0 (SDAS-9, clinical data); T1 (CTQ-SF, DERS, STAXI-2, AQ-RSV, ESI-BF); T2 (SDAS-9); T3 (DERS, STAXI-2, AQ-RSV, IPQ, interviews); and T4 (SDAS-9, DERS).

Measure	T0	T1	T2	T3	T4
Clinical and demographic data	X				
SDAS-9	X		X		X
CTQ-SF		X			
DERS		X		X	X
STAXI-2		X		X	
AQ-RSV		X		X	
ESI-BF		X			
IPQ				X	
Semi-structured interviews				X	

### Delivery of the VRAPT intervention

VRAPT was delivered by specially trained therapists (licensed psychologists and CBT therapists) at the current clinic. These therapists, with at least a step 1 psychotherapy education, were responsible for delivering psychotherapeutic treatment at the clinic and had considerable experience treating forensic psychiatric patients. All therapists received 16 h of training in the revised VRAPT protocol prior to participant enrolment. The training was provided by one of the VRAPT developers. In addition to this training, monthly group supervision was held by a licensed psychologist involved in the continued development of VRAPT (FRGM) throughout the intervention period. Ongoing clinical consultation was also available from the principal investigator (MW) whenever needed. Due to the COVID-19 pandemic, certain deviations from standard clinical procedures were necessary. Infection control measures were implemented in accordance with regional and national guidelines, including enhanced hygiene routines for VR equipment (e.g., disinfection of headsets and controllers between sessions), use of protective covers, and adjustments to session scheduling to reduce patient contact in shared spaces. When required, sessions were adapted or postponed to comply with safety regulations. These measures were taken to ensure both patient and staff safety without compromising the integrity of the intervention.

The intervention was delivered using the CleVR Social Worlds platform (see Figures 1–3 provide examples of virtual scenarios available within the platform), using Oculus Rift VR glasses, a high-performance laptop computer, a touch pad for controlling the virtual environment, and a microphone and headphones for communication between the therapist and participant. The platform enables therapists to dynamically control virtual avatars, verbal interactions, facial expressions, interpersonal distance, and scenario progression in real time according to the participant's therapeutic goals and emotional responses. During the virtual reality sessions, therapists gradually adjusted the level of interpersonal provocation and social complexity while guiding participants to recognize early emotional, cognitive, physiological, and behavioural signs of escalating anger. Participants practiced applying individualized emotion regulation strategies and alternative behavioural responses within these tailored virtual scenarios. Each session concluded with a structured therapeutic reflection aimed at consolidating learning and facilitating the transfer of acquired skills to everyday situations.

**Figure 1:**
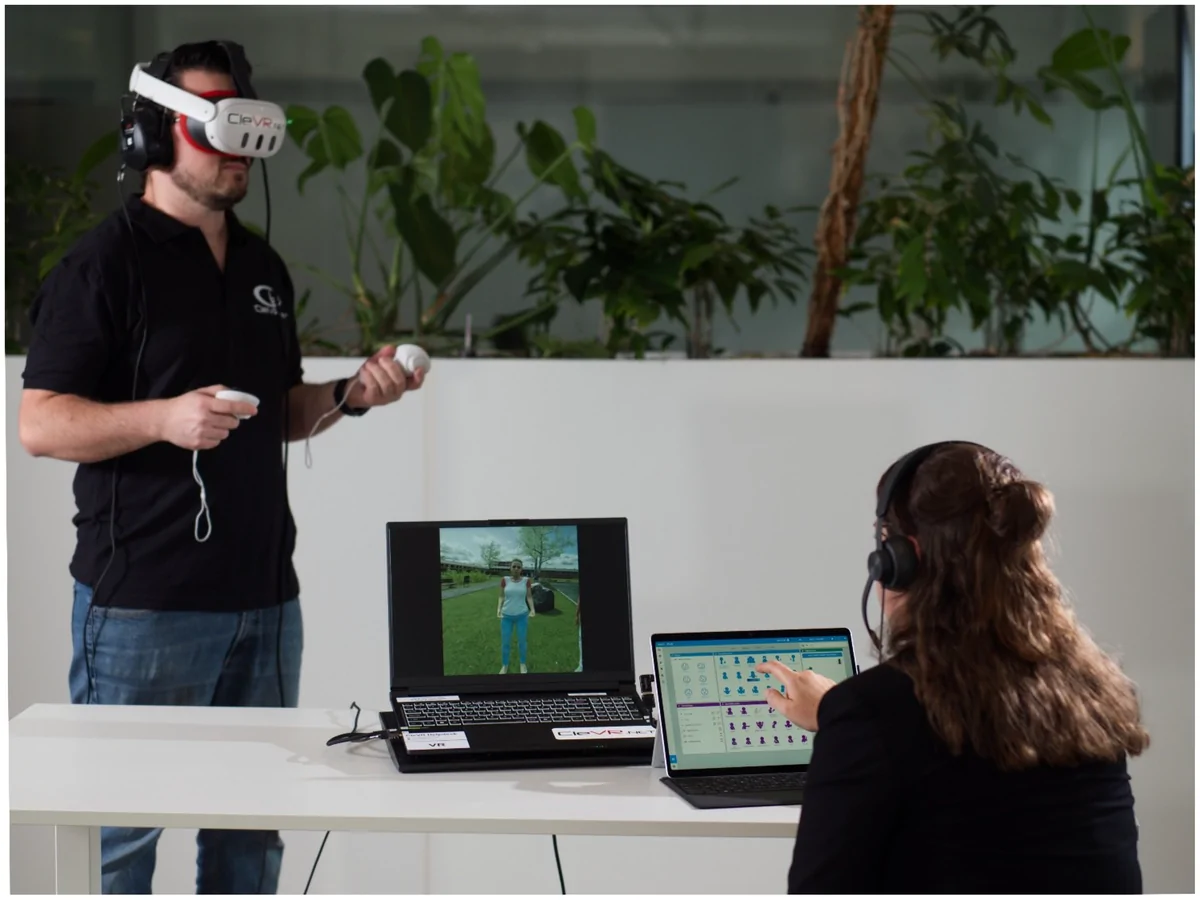
Illustrative representation of the VRAPT treatment setup using the CleVR social worlds platform. The image shows a typical setup in which the patient interacts with the virtual environment using a head-mounted display, while the therapist controls and adapts the virtual social scenario in real time through the therapist interface. Figure provided by CleVR.

**Figure 2:**
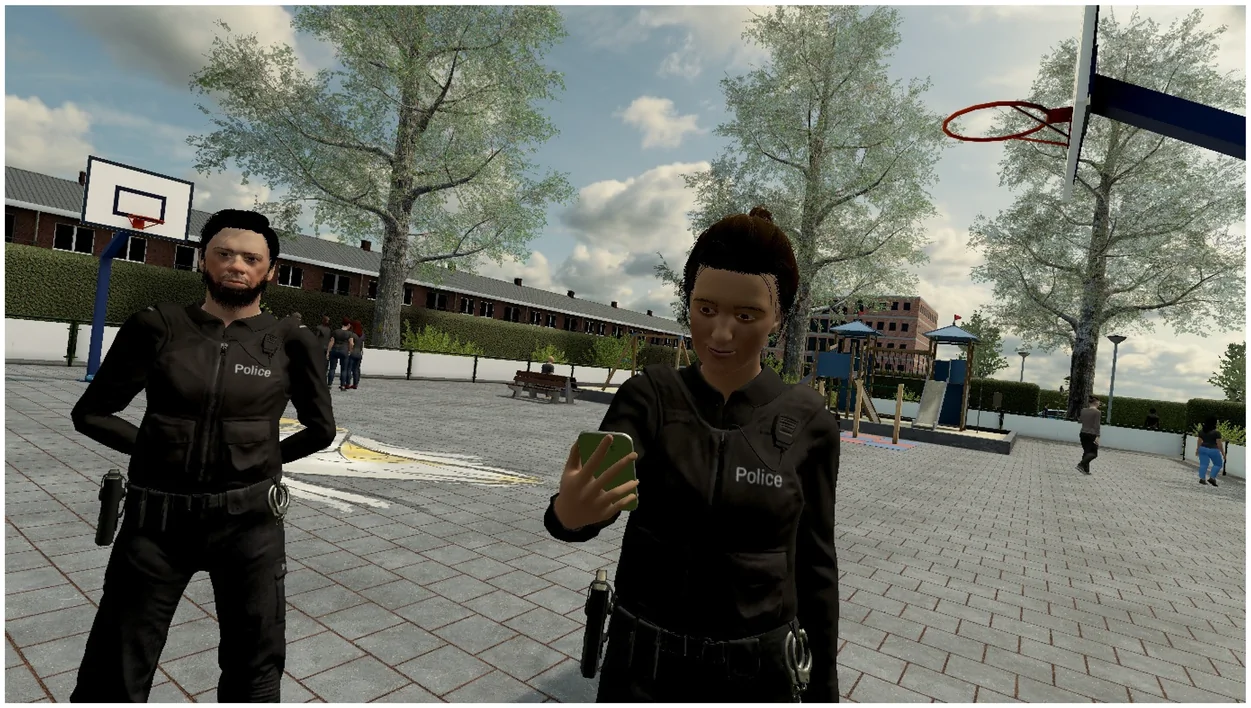
Example of a virtual interpersonal scenario available in the CleVR social worlds platform, depicting an interaction with two virtual police officers in a public outdoor setting, with other virtual characters in the surrounding environment. Figure provided by CleVR.

**Figure 3:**
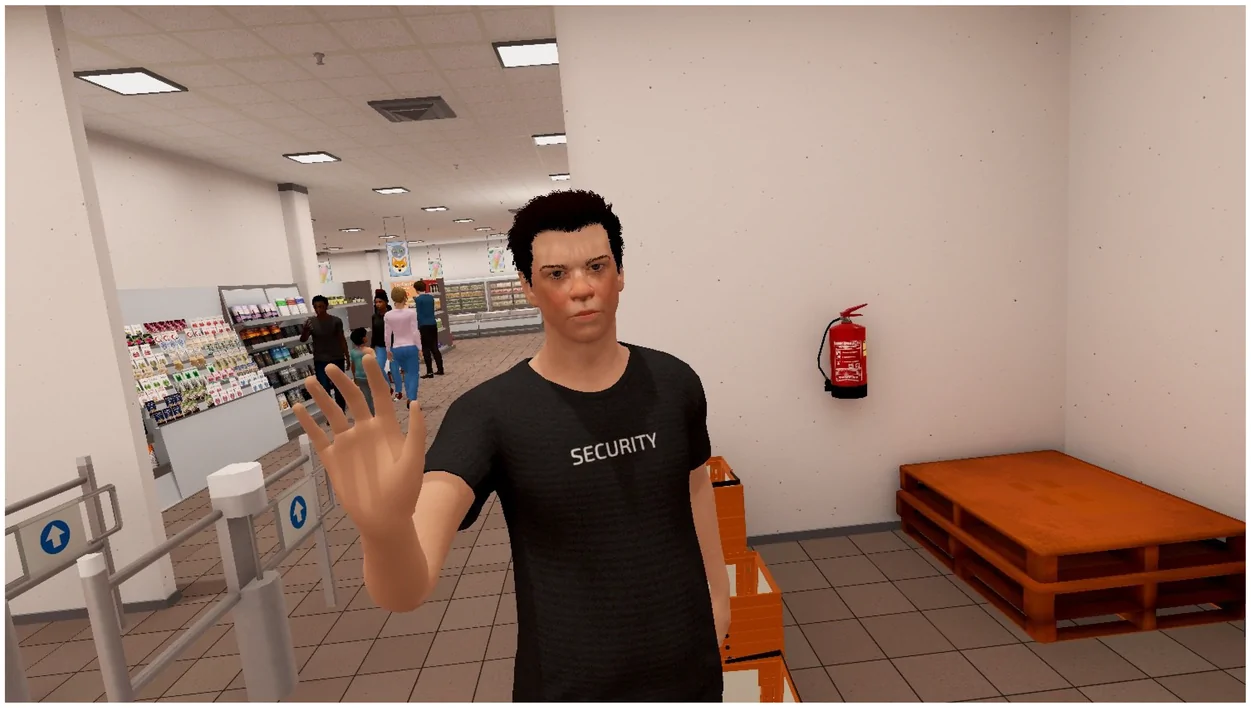
Example of a virtual interpersonal scenario available in the CleVR social worlds platform, depicting an interaction with a virtual security guard in a supermarket setting. Figure provided by CleVR.

Each session in the protocol followed a standardized structure consisting of: (a) a brief review of the previous session, (b) introduction of the corresponding treatment component within the VRAPT model, (c) discussion of the planned virtual scenario and therapeutic goals, (d) therapist-guided virtual reality exposure and skills practice, and (e) post-session reflection and evaluation of learning goals. To ensure treatment fidelity, therapists completed standardized session forms documenting treatment content and adherence to the protocol, which were subsequently reviewed for completeness by the research team. In addition, therapists submitted their session goals for supervision and feedback from clinical experts (FRGM and MW) throughout the intervention period.

### Fidelity in the VRAPT intervention

The revised VRAPT protocol (49) was designed as guidance for trainers and to ensure treatment integrity. Each session in the protocol followed the following format: (a) a short discussion of the previous session, (b) clarification of the corresponding step of the VRAPT model, (c) discussion of the planned VR scenario and therapeutic goals, (d) therapist-guided virtual reality exposure and skills practice, and (e) post-session reflection and evaluation of learning goals. Furthermore, during the intervention, trainers were required to complete session forms about treatment content and adherence to the protocol, which were checked for completeness by research assistants afterwards. Finally, for supervision purposes, therapists sent their learning goals to the clinical experts for feedback (FRGM, MW). Throughout the intervention, therapists also received ongoing clinical supervision from FRGM regarding treatment delivery, case formulation, and adherence to the therapeutic model. In addition, technical support related to the VR equipment and the CleVR Social Worlds platform was available whenever required. Technical issues were managed by FRGM, with additional support from the CleVR development team when required.

### Measures

Information on sociodemographic and clinical characteristics, as well as on current and previous violence, aggression, and criminality, was collected from three complementary sources: staff reports, participant self-reports, and file reviews. Data from file reviews was obtained using a structured data collection protocol, which included details on index offenses, prior offenses, violent incidents during forensic psychiatric care, and violence risk assessments. File-based information was gathered from mental healthcare medical records, all forensic psychiatric examinations (FPEs) conducted by the Swedish National Board of Forensic Medicine prior to sentencing, and official court verdicts.

The Social Dysfunction and Aggression Scale-9 [SDAS-9; (53)] is a widely used observer-rated instrument designed to measure aggression and social dysfunction in psychiatric and forensic settings and was also used as the primary outcome measure in the original VRAPT randomized controlled trial (42). SDAS-9 enables clinicians to assess the severity and frequency of aggressive behaviors across nine items and has been shown to be particularly useful for monitoring aggression over time, especially when evaluating the effects of psychopharmacological or behavioral interventions. In the present study, SDAS-9 was used as a staff-rated instrument to provide a systematic record of observed aggressive behaviors throughout the entire pilot period (T0–T4). Examples of behaviors assessed by the scale include irritability (e.g., difficulty controlling emotional reactions), negativism (e.g., refusal to cooperate), and directed verbal aggressiveness (e.g., personal insults). Each item was rated on a 5-point Likert scale ranging from “absent” to “severely present,” yielding a maximum total score of 45. Higher scores indicate greater levels of aggression and social dysfunction. SDAS-9 was completed on a weekly basis for all participants by specially trained staff on the treatment wards. Data collection began at T0 and continued throughout the study to T4, allowing for baseline and follow-up assessments of aggressive behavior before, during, and after the VRAPT intervention. Due to staff rotations based on institutional scheduling, different trained observers were at times responsible for the assessments of each participant. Interrater reliability was not assessed in this study. Previous research has demonstrated good convergent validity and clinical utility of SDAS-9 in evaluating aggressive behavior among forensic psychiatric patients (54). All staff observations were carried out by specially trained ward staff, supervised by the main author of this study.

The Aggression Questionnaire–Revised Swedish Version [AQ-RSV; (55)] was used as a self-reported, complementary measure of aggressive and hostile behaviors for all participants at T1 and T3. AQ-RSV is a Swedish adaptation of the original Aggression Questionnaire (56), which is widely used in aggression research. AQ-RSV comprises 29 items rated on a 4-point Likert scale, ranging from “least characteristic” to “most characteristic.” Items are grouped into four subscales: Physical Aggression, Verbal Aggression, Anger, and Hostility, as well as a Total Aggression Score, which aggregates the scores from all subscales. Higher scores indicate greater levels of self-reported aggression and hostility. Previous research has demonstrated good internal consistency for AQ-RSV (55).

The Difficulties in Emotion Regulation Scale [DERS; (57)] was used as a self-report measure of overall emotion regulation difficulties and was administered to all participants at T1, T3, and T4. DERS consists of 36 items rated on a 5-point Likert scale ranging from “Almost never” to “Almost always.” The scale includes six subscales assessing specific aspects of emotion dysregulation: (a) Non-Acceptance of Emotional Responses, (b) Difficulties Engaging in Goal-Directed Behavior, (c) Impulse Control Difficulties, (d) Lack of Emotional Awareness, (e) Limited Access to Emotion Regulation Strategies, and (f) Lack of Emotional Clarity. Higher scores indicate greater difficulties in emotion regulation. DERS has demonstrated strong psychometric properties in both clinical and non-clinical populations (57–59).

The State-Trait Anger Expression Inventory-2, Swedish version [STAXI-2S; (60)], a validated adaptation of the original STAXI-2 (61), was used as a self-report measure of participants’ experience, expression, and control of anger. STAXI-2S is a 57-item questionnaire that assesses anger across three core dimensions: (a) State Anger (the intensity of anger at a given moment), (b) Trait Anger (the general disposition to experience anger), and (c) Anger Expression (the frequency and manner in which anger is expressed). Items are rated on a 4-point Likert scale ranging from “Not at all” to “Almost always.” Because the meaning of higher scores differs across STAXI-2S subscales, with some reflecting greater anger-related difficulties and others reflecting more adaptive anger regulation, interpretation focused on individual subscale scores rather than an overall total score. The STAXI-2S was administered at T1 and T3. Previous studies have demonstrated good psychometric properties for the STAXI-2 and STAXI-2S, supporting their use in both general and clinical populations (60–62).

The Externalizing Spectrum Inventory–Brief Form [ESI-BF; (63)], a 160-item self-report instrument, was used to assess a broad spectrum of externalizing and aggressive behaviors, including callous-unemotional aggression. Items were rated on a 4-point scale ranging from “False” to “True” and were summed into three subscales: General Disinhibition, Callous-Aggression, and Substance Abuse. Higher scores on the ESI-BF and its subscales indicate greater levels of externalizing tendencies, including disinhibition, aggressive behavior, and substance-related problems. ESI-BF was administered to all participants at T1. A Swedish translation of the instrument was recently completed by the principal investigator of the present research program. ESI-BF has previously been used in both forensic and non-forensic populations and has demonstrated high reliability and predictive validity (64).

#### Sociodemographic and psychosocial background

Information on sociodemographic and psychosocial background was collected from two complementary sources: participant self-reports and case files. Data from case files were obtained using a structured data collection protocol designed to gather general sociodemographic information, such as age and gender, as well as psychosocial background variables (e.g., educational history, childhood circumstances including socio-economic status, institutionalization during childhood, work experience, and adverse childhood experiences). File-based information was extracted from mental healthcare medical records, all FPEs conducted for the participants, and court judgements. In the following section, the specific instruments used for data collection are described.

The Childhood Trauma Questionnaire – Short Form [CTQ-SF; (65)] was used as a screening tool to assess experiences of childhood abuse and neglect. CTQ-SF is designed for use with both adults and adolescents and evaluates five domains of childhood maltreatment: sexual abuse, physical abuse, emotional abuse, emotional neglect, and physical neglect. The questionnaire consists of 28 items, with five items corresponding to each subscale, and three additional items aimed at detecting potential minimization or denial of maltreatment experiences. Items are rated on a 5-point Likert scale ranging from 1 (“Never True”) to 5 (“Very Often True”). Higher scores on the subscales indicate greater reported exposure to childhood maltreatment within the respective domain. The Swedish version of the CTQ-SF has demonstrated comparable construct validity and internal consistency to the original version (66). The CTQ-SF was administered at T1.

#### Psychiatric characteristics

Information on current and previous mental disorders, assessments of intellectual functioning (when available), alcohol and substance use, prior contacts with the mental healthcare system, and other ongoing treatment interventions, including medication, was collected from case files using a structured data collection protocol. File-based information was obtained from mental healthcare medical records and all FPEs available at T0.

#### Experiences of the VRAPT intervention

The Igroup Presence Questionnaire [IPQ; (67)] was used to assess the participants’ sense of presence in the virtual environment at T3. IPQ consists of 17 items rated on a 7-point scale ranging from −3 (“completely disagree”) to +3 (“completely agree”), with item-specific anchors for some items, and has demonstrated good psychometric properties in previous research (68). In the present pilot descriptive case series, the IPQ was used to characterize participants’ sense of presence and immersive experience during the VRAPT intervention. A Swedish version of IPQ was recently translated by members of the research team and was used in a modified form, with the exclusion of items deemed irrelevant for the current study.

As the COVID-19 pandemic emerged during the course of the study, an ethical amendment was approved to allow assessment of participants’ COVID-19-related concerns. Consequently, the Fear of COVID-19 Scale [FCV-19S; (69)] was added to the assessment battery after study initiation. The FCV-19S is a seven-item self-report measure assessing emotional and physiological reactions associated with COVID-19, with higher scores indicating greater fear related to the pandemic. A Swedish translation of the FCV-19S, developed with permission from the original authors by FRGM, MW and KS were used in the present study. These measures were implemented to safeguard both patients and staff while preserving the integrity of the intervention. Participants generally reported low levels of COVID-19-related fear (FCV-19S: M = 12.29, SD = 6.91).

## Results

### Participant characteristics

All participants were receiving forensic psychiatric care under the Swedish Forensic Psychiatric Care Act with SDR. The mean duration of the current forensic psychiatric admission was 9.2 years (SD = 8.2), ranging from 3.0 to 23.0 years. Participants reported substantial psychosocial adversity during childhood and adolescence. Four participants (57.1%) reported having been bullied during their school years, four (57.1%) had witnessed violence between caregivers, four (57.1%) reported exposure to violence from caregivers, and four (57.1%) reported experiences of sexual victimization. Only two participants (28.6%) reported growing up with both parents throughout childhood, whereas five (71.4%) experienced the absence of one or both caregivers during childhood. Three participants (42.9%) had been placed outside the home during childhood. Regarding psychiatric history, four participants (57.1%) had previous contact with child and adolescent mental health services, three (42.9%) had previous contact with adult psychiatric services, three (42.9%) had received substance abuse treatment, and two (28.6%) had previously received forensic psychiatric care. All participants received psychotropic medication during the study period, and five participants (71.4%) also received individual psychological treatment. Structured violence risk assessments had been conducted for all participants within the previous two years.

The sample was characterized by severe and complex psychiatric morbidity. Most participants met criteria for schizophrenia spectrum disorders, often in combination with neurodevelopmental disorders, personality pathology, affective symptoms, and substance use disorders. Together with the high prevalence of childhood adversity and previous psychiatric treatment, these characteristics reflect the complexity typically observed in forensic psychiatric populations. Childhood trauma was common in the sample. Mean CTQ-SF scores were 10.14 (SD = 5.08) for emotional abuse, 8.57 (SD = 4.54) for physical abuse, 10.14 (SD = 8.08) for sexual abuse, 12.57 (SD = 4.24) for emotional neglect, and 10.14 (SD = 3.98) for physical neglect. Baseline ESI-BF scores indicated elevated externalizing characteristics in the sample, with mean scores of 30.14 (SD = 10.53) for General Disinhibition, 19.29 (SD = 13.61) for Callous-Aggression, and 27.43 (SD = 9.05) for Substance Abuse. Participants reported low to moderate levels of COVID-19-related fear (FCV-19S: M = 12.29, SD = 6.91).

### Aggressive behaviour (SDAS-9)

Given the case-series design, particular emphasis was placed on examining individual patterns of change over time. Figure 4 displays weekly SDAS-9 trajectories for each participant across the pre-treatment, VRAPT, and follow-up phases, providing an overview of both within-participant change and between-participant variability. Because treatment duration varied across participants, the number of observations within the VRAPT phase differed between cases, which is reflected in the differing lengths of the treatment trajectories shown in Figure 4. Visual inspection revealed substantial heterogeneity in the development of aggressive behaviour over time. Three participants (P2, P3, and P7) demonstrated an overall reduction in SDAS-9 scores from the pre-treatment phase to follow-up. Participant P3 showed the most pronounced improvement, with aggressive behaviour decreasing progressively and reaching near-zero levels during follow-up. Participant P2 also demonstrated consistently low aggression scores throughout the study, with a further reduction during follow-up. Participant P7 initially displayed the highest levels of aggressive behaviour, including a marked peak during the intervention period, followed by a gradual decline over time. In contrast, two participants (P1 and P6) showed increased SDAS-9 scores during follow-up compared with baseline. Participant P1 exhibited relatively low levels of aggressive behaviour during the pre-treatment phase but showed elevated scores during follow-up. Participant P6 demonstrated a more variable trajectory, with a reduction during the intervention phase followed by a subsequent increase during follow-up. Participants P4 and P5 showed relatively stable trajectories across study phases. Although some fluctuations were observed, no substantial or sustained changes in aggressive behaviour were evident. At the group level, mean SDAS-9 scores were 2.56 during the pre-treatment phase, 2.40 during the VRAPT phase, and 2.48 during follow-up. These descriptive findings indicate modest changes in aggressive behaviour over time, although considerable between-participant variability was observed. An exploratory Wilcoxon signed-rank test comparing pre-treatment and follow-up SDAS-9 scores did not indicate a statistically significant difference at the group level (V = 18, *p* = .578).

**Figure 4:**
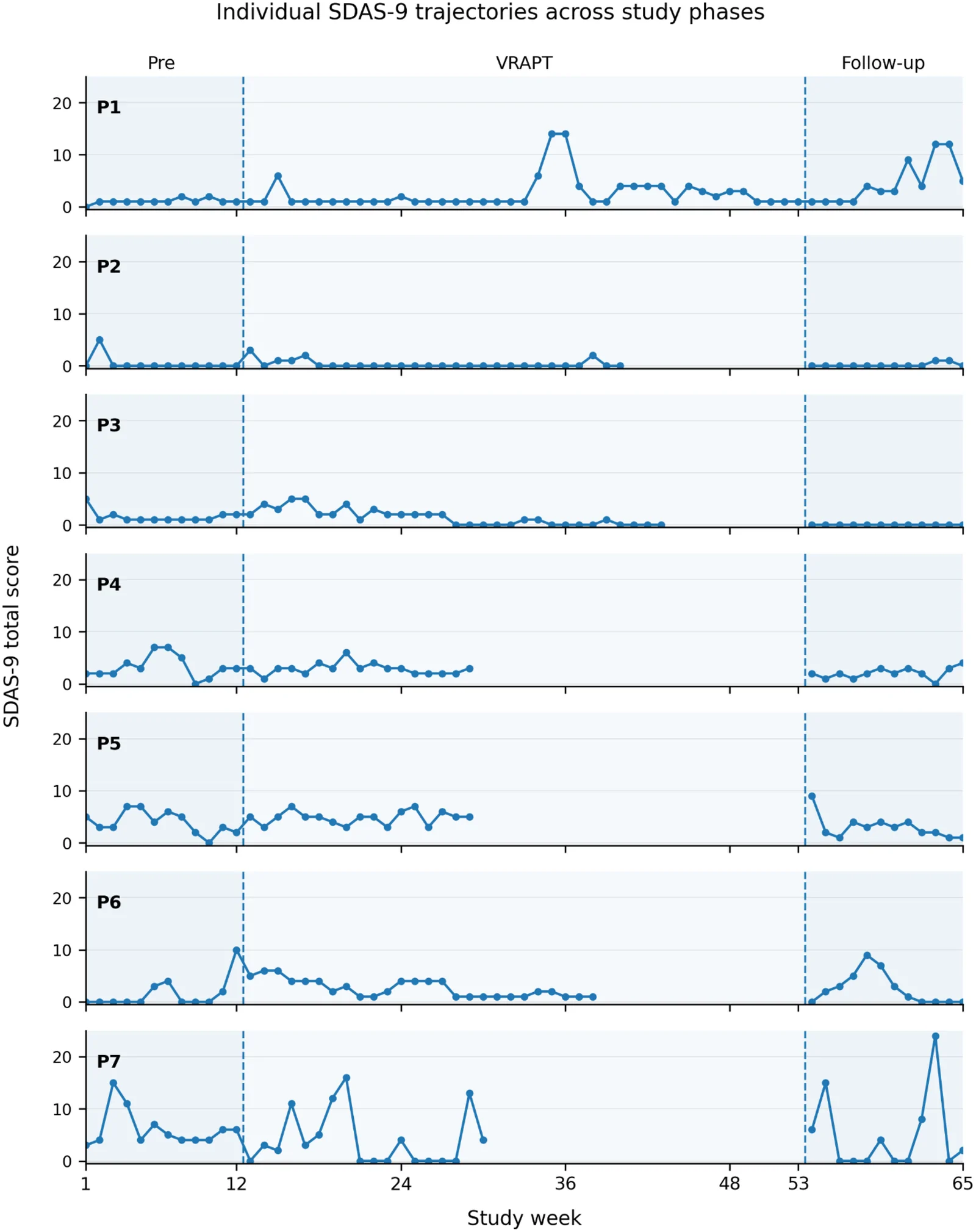
Individual SDAS-9 trajectories across study phases. Weekly SDAS-9 total scores for each participant during the pre-treatment (12 weeks), VRAPT intervention (up to 41 weeks), and follow-up (12 weeks) phases. Dashed vertical lines indicate transitions between study phases. Considerable heterogeneity was observed across participants, with substantial variation in individual SDAS-9 trajectories across study phases.

### Difficulties in emotion regulation (DERS)

Changes in emotion regulation were assessed using DERS (see Table 2) at baseline (T1), post-treatment (T3), and follow-up (T4). At group level, mean DERS total scores decreased from 71.86 (SD = 18.52) at baseline to 69.00 (SD = 20.79) post-treatment and 66.14 (SD = 17.54) at follow-up. Visual inspection showed heterogeneous individual trajectories, with most participants demonstrating reductions in emotion regulation difficulties over time, whereas one participant showed a marked increase in DERS scores during the intervention period (Figure 5).

**Table 2:** Descriptive statistics for DERS total scores.

Participant	T1	T3	T4	Change T1–T4
P1	95	75	70	−25
P2	65	64	64	−1
P3	54	40	40	−14
P4	64	52	51	−13
P5	49	104	93	44
P6	93	66	65	−28
P7	83	82	80	−3

**Figure 5:**
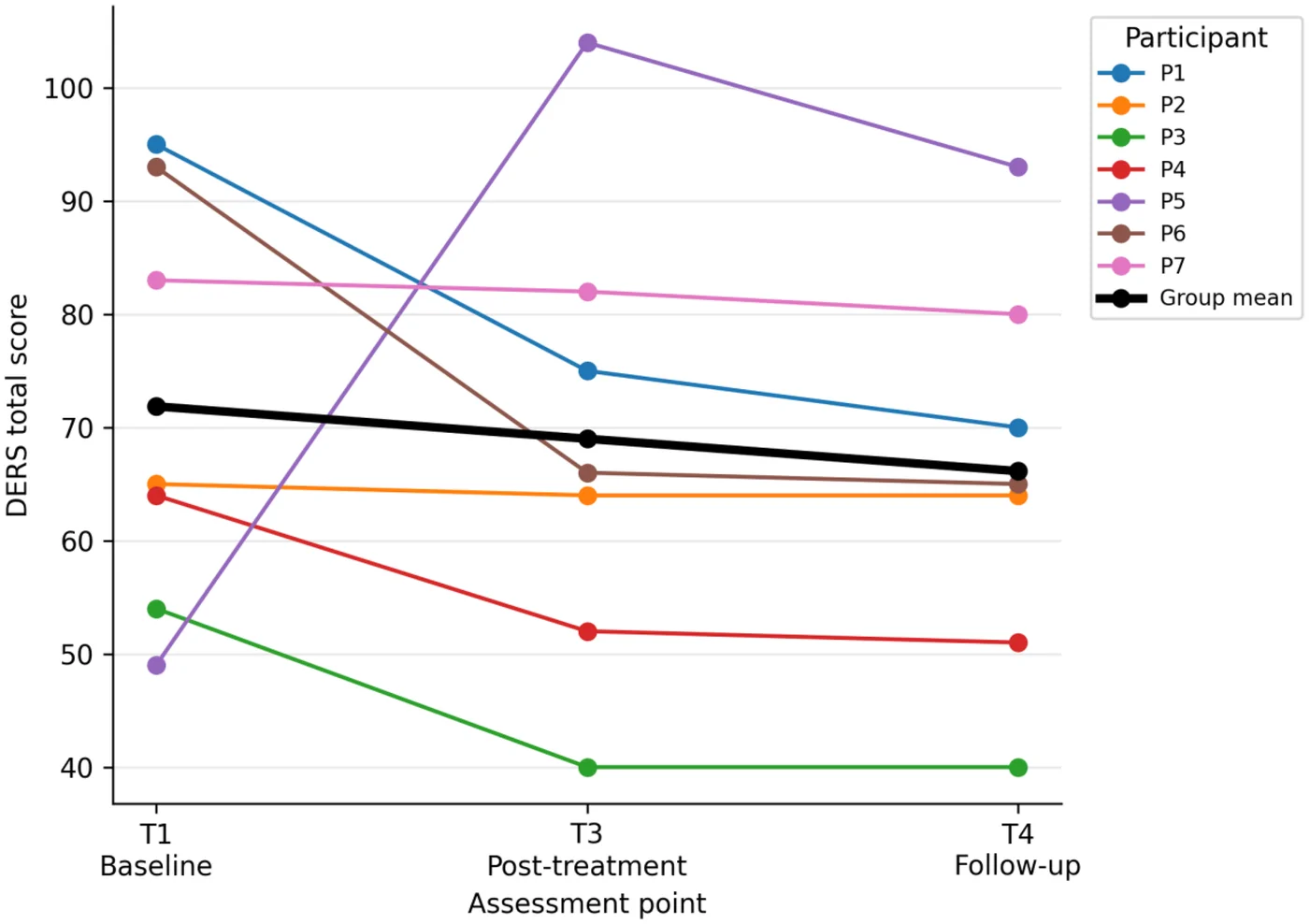
Total difficulties in emotion regulation scale (DERS) scores at baseline (T1), post-treatment (T3), and follow-up (T4) for each participant. The black line represents the group mean. Higher scores indicate greater difficulties in emotion regulation.

Subscale analyses (Table 3) indicated the largest reductions in the Strategies and Clarity domains. Mean Strategies scores decreased from 16.14 (SD = 5.76) at baseline to 13.43 (SD = 4.65) at follow-up, whereas Clarity scores decreased from 8.71 (SD = 4.42) to 6.29 (SD = 1.25). Smaller changes were observed for Awareness and Impulse difficulties, whereas Nonacceptance and Goals scores remained relatively stable across assessment points. Taken together, these descriptive findings indicate that several participants showed reductions in emotion regulation difficulties over time, particularly in the Strategies and Clarity domains. However, considerable between-participant variability was observed, and the findings should be interpreted cautiously given the exploratory pilot descriptive case-series design.

**Table 3:** DERS subscale means and standard deviations across assessment points.

Subscale	T1 mean (SD)	T3 mean (SD)	T4 mean (SD)
Nonacceptance	9.57 (3.99)	10.71 (5.91)	10.14 (5.24)
Goals	14.71 (5.85)	15.43 (5.88)	15.43 (6.11)
Impulse	10.14 (4.71)	10.71 (5.65)	9.57 (3.69)
Awareness	12.57 (4.35)	12.00 (2.52)	11.29 (1.98)
Strategies	16.14 (5.76)	13.71 (4.99)	13.43 (4.65)
Clarity	8.71 (4.42)	6.43 (1.51)	6.29 (1.25)

Descriptive changes from baseline (T1) to post-treatment (T2) were observed in several anger-related and aggressive tendencies (see Table 4). On the STAXI-2, mean scores decreased for Angry Temperament, Angry Reaction, and the Anger Expression Index, while Anger Control-Out and Anger Control-In increased. The largest change was observed for Anger Control-In (+4.58 points), indicating higher Anger Control-In scores at post-treatment. The Anger Expression Index decreased by 5.86 points, reflecting lower Anger Expression Index scores at post-treatment. Because the STAXI-2 includes subscales where higher scores may indicate either improvement or deterioration, the total score is difficult to interpret. Consequently, interpretation focused on the clinically relevant subscales, which showed a pattern consistent with changes in anger regulation across participants. On AQ-RSV, reductions were observed for Physical Aggression, Verbal Aggression, and Anger. The AQ-RSV total score decreased from 81.71 (SD = 22.17) at baseline to 73.86 (SD = 15.83) post-treatment, representing a mean reduction of 7.85 points. Hostility remained relatively stable. Overall, descriptive changes in AQ-RSV scores were consistent with lower self-reported aggression in several participants, although individual variability remained evident.

**Table 4:** Changes in self-reported anger and aggression from baseline (T1) to post-treatment (T2). STAXI-2 and AQ-RSV Outcomes (T1–T2).

Outcome	T1 mean (SD)	T2 mean (SD)	Change (T2-T1)
Angry Temperament	7.00 (4.00)	5.43 (1.62)	−1.57
Angry Reaction	7.86 (3.58)	6.14 (2.19)	−1.72
Anger Expression-Out	12.71 (5.54)	12.43 (2.57)	−0.28
Anger Expression-In	18.14 (6.38)	19.43 (5.59)	+1.29
Anger Control-Out	23.86 (5.26)	26.14 (4.33)	+2.28
Anger Control-In	19.71 (7.76)	24.29 (8.54)	+4.58
Anger Expression Index	35.29 (18.15)	29.43 (15.81)	−5.86
STAXI-2 Total Score	108.00 (11.88)	112.57 (11.87)	+4.57
Physical Aggression	23.29 (7.43)	21.00 (6.63)	−2.29
Verbal Aggression	14.43 (2.82)	12.00 (2.94)	−2.43
Anger	20.86 (6.69)	17.43 (6.09)	−3.43
Hostility	23.14 (5.59)	23.43 (4.97)	+0.29
AQ-RSV Total Score	81.71 (22.17)	73.86 (15.83)	−7.85

### Presence in the virtual environment (IPQ)

Participants’ experience of presence within the virtual environment was assessed at post-treatment (T3) using the Igroup Presence Questionnaire (IPQ; see Table 5). Overall, participants reported a moderate to high sense of presence while interacting with the VRAPT environments. Mean scores indicated moderate levels of Spatial Presence (M = 3.86, SD = 0.69), Experienced Realism (M = 3.43, SD = 0.79), and General Presence (M = 2.86, SD = 1.57). Participants also reported moderate involvement in the virtual scenarios (M = 11.29, SD = 5.97), although substantial variability was observed across individuals. The mean IPQ total score was 5.43 (SD = 0.98), with scores ranging from 4 to 7. Taken together, these findings indicate moderate to high levels of perceived presence across participants, although individual variability was observed.

**Table 5:** Presence in the virtual environment assessed with the iGroup presence questionnaire (IPQ).

IPQ	Mean (SD)	Range
Spatial presence	3.86 (0.69)	3–5
Involvement	11.29 (5.97)	5–19
Experienced realism	3.43 (0.79)	3–5
General presence	2.86 (1.57)	1–5
IPQ total score	5.43 (0.98)	4–7

## Discussion

The present pilot descriptive case series study described the clinical application of the revised VRAPT protocol and individual trajectories in a sample of seven forensic psychiatric patients receiving compulsory forensic psychiatric care in Sweden. Overall, heterogeneous patterns were observed across measures of emotion regulation, anger, self-reported aggression, and staff-rated aggressive behaviour. Several participants showed reductions in some measures over time, whereas others showed limited change or increases, and no statistically significant group-level change was observed in staff-rated aggression. Participants also reported moderate to high levels of perceived presence in the virtual environment. These findings indicate that participants generally reported a moderate to high sense of presence while interacting with the VRAPT environments. This is an important finding because a sense of presence has been proposed as an important prerequisite for experiential learning in virtual reality interventions (70). Furthermore, immersive virtual environments may enhance the plausibility of virtual experiences, that is, the extent to which virtual events are experienced as actually occurring and warrant genuine emotional and behavioural responses (71). When individuals perceive virtual environments as meaningful and believable, emotional and behavioural responses are more likely to resemble those occurring in real-life situations, thereby enhancing opportunities for skills acquisition and transfer to everyday contexts (70–72). The observed levels of presence, despite the considerable clinical heterogeneity of the sample, are consistent with the theoretical rationale underlying VRAPT, which proposes that emotionally meaningful virtual experiences facilitate experiential learning and the practice of emotion regulation strategies. The observed dropout rate nevertheless suggests that maintaining treatment engagement remains an important challenge for future studies (73).

An important observation in the present case series study was the heterogeneity in trajectories of emotion regulation difficulties across participants. While group-level changes in DERS scores were modest, several participants showed reductions over time, whereas others showed little change or increases. Difficulties in emotion regulation are considered a central risk factor for reactive aggression and violent behavior (74, 75), and impairments in emotional awareness and impulse control are commonly reported among forensic psychiatric populations (76, 77). These descriptive findings are consistent with the theoretical rationale underlying VRAPT, which aims to increase awareness of emotional and physiological reactions in provocative social situations while providing opportunities to practice alternative behavioural responses in a safe environment. Given the descriptive design, these observations should be interpreted cautiously and warrant further evaluation in controlled studies.

Descriptive changes in the STAXI-2 subscales provided additional information on patterns of anger-related processes across participants. Participants showed reductions in Angry Temperament, Angry Reaction, and the Anger Expression Index, together with increases in Anger Control-Out and Anger Control-In. Overall, the pattern of change across the STAXI-2 subscales was more consistent with changes in anger regulation than with changes in the experience of anger itself. This distinction is important because the goal of aggression prevention interventions is not necessarily to eliminate anger, but rather to improve individuals’ ability to manage and express anger in adaptive ways. The observed increases in anger control scores are consistent with this theoretical perspective and support further investigation in controlled studies.

Descriptive changes in AQ-RSV scores indicated lower self-reported aggression in several participants. Reductions were observed in Physical Aggression, Verbal Aggression, and Anger, while Hostility remained relatively stable. The AQ-RSV total score decreased by nearly eight points on average, although individual variability was observed across participants. The relative stability of Hostility scores may warrant further investigation to determine whether cognitive aspects of aggression respond differently from behavioural and emotional dimensions, as suggested by previous research on the relative persistence of entrenched cognitive schemas (78).

The SDAS-9 trajectories provided a more nuanced picture of changes in staff-rated aggressive behaviour over time. Although overall group-level changes were limited, individual trajectories revealed substantial heterogeneity. Some participants showed reductions in aggressive behaviour whereas others showed little change or fluctuating patterns over time. Such variability is common in forensic psychiatric populations, which are characterized by considerable differences in diagnosis, cognitive functioning, trauma exposure, treatment history, and risk profiles (79–81). These findings highlight the importance of examining patterns of change beyond group averages, particularly in small samples and early-stage intervention research.

The baseline characteristics of the participants underscore the complexity of the population receiving VRAPT. The sample reported extensive childhood adversity, including emotional abuse, neglect, exposure to violence, bullying, and sexual victimization. These findings are consistent with previous studies demonstrating high rates of adverse childhood experiences among forensic psychiatric patients (82). Childhood trauma has repeatedly been linked to difficulties in emotion regulation, aggressive behavior, interpersonal problems, and increased risk of violent offending (79). From this perspective, the observed heterogeneity in trajectories of emotion regulation and anger-related measures should be interpreted within the broader clinical context of extensive childhood adversity, as these mechanisms are often rooted in early developmental experiences.

Participants also displayed elevated levels of externalizing psychopathology, including disinhibition, callous-aggressive traits, and substance-related problems. Such characteristics are strongly associated with persistent antisocial behavior and violence risk (83). Together with the extensive childhood adversity observed in the sample, these characteristics provide important clinical context for interpreting the marked heterogeneity in individual trajectories. Individuals with more pronounced externalizing tendencies may require longer or more intensive interventions to achieve behavioural change, a possibility that should be examined in future studies (84).

An important characteristic of VRAPT is its ability to provide repeated, therapist-guided exposure to emotionally provocative interpersonal situations within a controlled and safe virtual environment. Unlike traditional role-play exercises, VRAPT allows therapists to tailor these emotionally provocative scenarios to the individual's triggers and therapeutic goals while maintaining a high degree of control and safety (85). The moderate to high levels of perceived presence reported by participants are consistent with the assumption that the virtual environments were experienced as sufficiently realistic to support emotionally relevant interactions (86). Such immersion may facilitate emotional activation during treatment, thereby creating opportunities for experiential learning that are difficult to achieve through conventional verbal interventions alone. This mechanism has been proposed as particularly valuable in forensic psychiatric settings, where transferring therapeutic skills from the therapy room to real-world situations can be challenging. The present findings are consistent with this theoretical framework and with recent qualitative evidence suggesting that VR-based role-play is perceived by therapists and ward staff as a valuable component of aggression treatment in forensic psychiatric settings (52). Taken together, these descriptive observations provide a basis for future controlled studies evaluating the revised VRAPT protocol as a potential adjunctive intervention for the treatment of aggression in forensic psychiatric care.

### Limitations

Several limitations should be considered when interpreting the findings of this study. First, the study included a small sample of only seven participants from a single maximum-security forensic psychiatric clinic. Although case-series designs are valuable for exploring novel interventions in complex clinical populations, the limited sample size reduces statistical power and restricts the generalizability of the findings. The findings should therefore be regarded as descriptive and hypothesis-generating, providing a basis for future controlled studies rather than conclusions regarding treatment effectiveness. Second, the absence of a control group precludes conclusions regarding whether the observed changes were attributable to VRAPT. Changes observed over time may have been influenced by factors other than the intervention itself, including ongoing psychiatric treatment, medication adjustments, maturation, regression to the mean, or changes in the ward environment. Although efforts were made to document concurrent treatments, it is not possible to determine the specific contribution of VRAPT to the observed patterns of change. Third, the study was conducted during the COVID-19 pandemic, which affected both clinical practice and research procedures. Infection-control measures occasionally required adjustments to treatment delivery and scheduling, and the broader psychosocial impact of the pandemic may have influenced participants’ emotional functioning and behavior. To better understand these effects, the Fear of COVID-19 Scale (FCV-19S) was added to the assessment battery through an ethical amendment after study initiation. Nevertheless, the pandemic represents a contextual factor that may limit the comparability of the observed findings with those obtained under more typical clinical circumstances. Fourth, aggressive behavior was assessed using the SDAS-9, which relied on ratings completed by ward staff. Given the rotating nature of staffing within the clinic, multiple raters contributed to the assessments over time. Inter-rater reliability was not formally evaluated, and differences in staff perceptions and rating practices may therefore have influenced the scores. Although the SDAS-9 provides valuable information regarding observable aggression, such variability introduces an additional source of measurement error. Furthermore, the highly structured and closely supervised nature of a maximum-security forensic psychiatric setting may reduce the sensitivity of the SDAS-9 to detect changes in observable aggressive behaviour over time, as opportunities for overt aggressive behavior are limited and incidents may be prevented through routine clinical management. Fifth, the study relied heavily on self-report measures to assess emotion regulation, anger, aggression, trauma history, and externalizing traits. Self-report instruments may be influenced by social desirability, limited insight, recall bias, or response tendencies. These concerns may be particularly relevant in forensic psychiatric populations, where participants may have varying levels of cognitive functioning, self-awareness, and capacity to accurately report their experiences and behaviours. Sixth, several participants withdrew from the study before completing the intervention or follow-up assessments. Recruitment and retention challenges reduced the final sample size and may have introduced selection bias, as individuals who completed treatment may differ systematically from those who discontinued participation. Consequently, the observed patterns of change may not be representative of all patients who initiated the intervention. At the same time, the dropout rate provides important information regarding treatment engagement and retention in a maximum-security forensic psychiatric setting. Future studies should investigate factors associated with treatment engagement, acceptability, and retention. Finally, although descriptive changes were observed across several quantitative measures, the study was not designed to evaluate long-term outcomes such as recidivism, institutional violence, or sustained behavioral change after discharge. Future research should include larger samples, longer follow-up periods, and multi-site recruitment to determine whether the observed patterns of change translate into meaningful reductions in aggression and violence risk over time. Given the substantial heterogeneity of forensic psychiatric patients, high-quality single-case experimental designs may also provide valuable insights into individual treatment responses and the mechanisms underlying therapeutic change. Despite these limitations, this study provides the first detailed description of the implementation of the revised VRAPT protocol within a Swedish maximum-security forensic psychiatric setting. The findings contribute descriptive clinical observations that may inform the design of future randomized controlled trials and high-quality single-case experimental studies.

## Conclusion

This pilot descriptive case series provides the first detailed description of the implementation of the revised Virtual Reality Aggression Prevention Training (VRAPT) protocol within a Swedish maximum-security forensic psychiatric setting. Across multiple measures, heterogeneous patterns of change were observed in emotion regulation, anger, self-reported aggression, and staff-rated aggressive behaviour. Although several participants showed favourable changes over time, others demonstrated little change or fluctuating trajectories. Given the uncontrolled design, these observations should be interpreted cautiously and cannot be attributed to the VRAPT intervention. The findings are consistent with the theoretical rationale underlying VRAPT, which proposes that immersive virtual reality can provide a controlled environment for practicing emotional awareness, emotion regulation, and alternative behavioural responses during emotionally provocative interpersonal situations. Participants also reported moderate to high levels of perceived presence, suggesting that the virtual environments were experienced as sufficiently realistic to support experiential learning. Overall, this study establishes a clinical foundation for evaluating the revised VRAPT protocol as a potential adjunctive intervention for the treatment of aggression in forensic psychiatric care. The heterogeneity of treatment responses highlights the importance of considering individual clinical characteristics, treatment needs, and contextual factors when designing and evaluating VR-based interventions in forensic settings. Future research should include larger controlled studies, extended follow-up periods, and multi-site recruitment to determine whether the observed patterns of change are attributable to VRAPT and whether they translate into meaningful reductions in aggression and violence risk. Given the complexity of forensic psychiatric populations, high-quality single-case experimental designs may be particularly valuable for identifying which patients benefit most from VRAPT and for understanding the mechanisms underlying therapeutic change.

## Data Availability

The raw data supporting the conclusions of this article will be made available by the authors, without undue reservation.

## Supplementary material

Data Sheet 1 Swedish version of the Fear of Coronavirus-19 Scale (FCV-19S).
