# Supplementary material for: Virtual reality-assisted psychological treatment of aggression for violent offenders: a pilot descriptive case series in maximum-security forensic psychiatry
Source: Front Health Serv. 2026 Sep 29;6:1920018. doi: 10.3389/frhs.2026.1920018 (PMC13658505; doi:10.3389/frhs.2026.1920018)
Supplement: Supplementary file 1 [file Datasheet1.pdf]

Fear of Coronavirus-19 Scale (FCV-19S)

Ahorsu, Lin, Imani, Saffari, Griffiths, & Pakpour (2020) [1]

Svensk översättning med tillstånd av originalförfattare:

Fernando González Moraga, Märta Wallinius & Kristina Sygel
Rättspsykiatriska regionkliniken, Växjö

25 november, 2020

	Stämmer inte alls	Stämmer inte	Varken stämmer eller stämmer inte	Stämmer	Stämmer precis
1. Jag är väldigt rädd för covid-19					
2. Det gör mig obekväm att tänka på covid-19					
3. Mina händer blir svettiga när jag tänker på covid-19					
4. Jag är rädd för att förlora mitt liv på grund av covid-19					
5. När jag tittar på nyheter och berättelser om covid-19 på sociala medier blir jag nervös eller orolig					
6. Jag kan inte sova eftersom jag oroar mig för att få covid-19					
7. Mitt hjärta rusar eller slår snabbt när jag tänker på att få covid-19					

Deltagarna anger sin nivå av överensstämmelse med påståendena med hjälp av en 5-gradig likertskala. Svarsalternativen är ”stämmer inte alls”, “stämmer inte”, “varken stämmer eller stämmer inte”, “stämmer” och “stämmer precis”. Lägsta möjliga poäng för varje påstående är 1 och maximala är 5. En totalpoäng (från 7 till 35 poäng) beräknas genom att lägga samman poängen för varje påstående. Ju högre poäng, desto större rädsla för covid-19.

[1]Ahorsu, D. K., Lin, C. Y., Imani, V., Saffari, M., Griffiths, M. D., & Pakpour, A. H. (2020). The fear of COVID-19 scale: development and initial validation. International journal of mental health and addiction.
